# Draft Genome Sequence of Hydrocarbon-Degrading *Pseudomonas putida* Strain KG-4, Isolated from Soil Samples Collected from Krishna-Godavari Basin in India

**DOI:** 10.1128/genomeA.00590-15

**Published:** 2015-06-04

**Authors:** Chhavi Dawar, Ramesh K. Aggarwal

**Affiliations:** Centre for Cellular and Molecular Biology (CSIR-CCMB), Tarnaka, Hyderabad, India

## Abstract

We report here the 5.58-Mb draft genome of *Pseudomonas putida* strain KG-4 obtained from the oil fields of the Krishna-Godavari basin, Andhra Pradesh, India. The genome sequence is expected to facilitate identification and understanding of genes associated with hydrocarbon metabolism, which can help in developing strategies for managing oil spills and bioremediation.

## GENOME ANNOUNCEMENT

*Pseudomonas putida* is a Gram-negative saprotrophic bacterium found in soil and aqueous habitats. It has a versatile metabolism and has been reported to have important functions in biodegradation and biotransformation of aliphatic and aromatic compounds (1–3). Here, we report the draft genome sequence of *P. putida* strain KG-4, which was collected from soil (at a depth of 1.0 m) from the east Godavari subbasin of the Krishna-Godavari (K-G) basin as part of a pilot study to understand the bacterial diversity in the oil fields of Krishna-Godavari basin, in Andhra Pradesh, India. The isolate identity was confirmed to the species level by typing the 16S rRNA.

We sequenced the genome of *Pseudomonas putida* KG-4 using the Roche 454 (FLX Titanium) pyrosequencing platform, which yielded a total of 107,387,548 bases in 286,508 reads. GS De Novo Assembler (version 2.8) was used to assemble the reads, which generated a total of 91 contigs with a median contig sequence size (*N*_50_) of 0.15 Mb and a longest contig size of 0.78 Mb. Of all the bases, 98.68% were assembled into a 5.58-Mb draft genome with a G+C content of 63%. These contigs were submitted to both the Rapid Annotations using Subsystem Technology (RAST) server (4) and the NCBI Prokaryotic Genome Annotation Pipeline (PGAP) for annotation. Metabolic pathways were examined through the KEGG Automatic Annotation Server (KAAS) (5).

NCBI annotation produced a total of 5,068 genes, 4,920 coding sequences (CDS), 76 pseudogenes, 5 rRNAs, 65 tRNAs, and 2 noncoding RNAs (ncRNAs). RAST gave comparable results, with 520 subsystems. The RAST server annotated 113 functional genes for the subsystem of metabolism of aromatic compounds, including subcategories for peripheral pathways, catabolism of aromatic compounds, metabolism of central aromatic intermediates, and metabolism of aromatic compounds. According to KEGG analysis, several pathways, *viz.,* bisphenol degradation, limonene and pinene degradation, geraniol degradation, chloroalkane/alkene degradation, chlorocyclohexane and chlorobenzene degradation, styrene degradation, atrazine degradation, caprolactam degradation, dioxin degradation, naphthalene degradation, fatty acid degradation, and polycyclic aromatic hydrocarbon degradation, were found to be unique in *P. putida* KG-4 compared to those in other *P. putida* genomes (KT2440, GB1, and F1), in addition to the common pathways, such as those for benzoate, toluene, and ethylbenzene degradation. Degradation pathways of aminobenzoate, dioxin, bisphenol, chloroalkane, and chloroalkene found in the previously reported naphthalene-utilizing strain *P. putida* OUS82 (6) were also found in KG-4. Similarly, bisphenol degradation, polycyclic aromatic hydrocarbon degradation, and ethylbenzene degradation were observed in both abiotic stress-tolerant *P. putida* MTCC5279 (7) and KG-4.

With the large diversity of pathway enzymes we predicted in strain KG-4, it becomes evident that this strain can be an important resource for biotechnological applications like industrial wastewater management and oil spill degradation. Thus, this draft genome sequence may be instrumental in the identification and characterization of various genes encoding important microbial enzymes that can play significant roles in bioremediation.

### Nucleotide sequence accession number. 

This whole-genome shotgun project has been deposited at DDBJ/EMBL/GenBank under the accession number AYRY00000000. The version described in this paper is the first version.
